# Molecular pharmacokinetic mechanism of quercetin-encapsulated polymeric micelles in alleviating cisplatin-induced nephrotoxicity and enhancing antineoplastic effects

**DOI:** 10.3389/fphar.2025.1590688

**Published:** 2025-06-09

**Authors:** Tangna Hao, Xiaokui Huo, Zhen Li, Changyuan Wang, Sha Wu, Anni Song, Fengyu Zhang, Kexin Liu

**Affiliations:** ^1^ Institute of Integrative Medicine, Dalian Medical University, Dalian, China; ^2^ Provincial Key Laboratory for Pharmacokinetics and Transport, Liaoning Dalian Medical University, Dalian, China; ^3^ Department of Pharmacy, The Second Affiliated Hospital of Dalian Medical University, Dalian, China; ^4^ Pharmaceutical Research Center, Second Affiliated Hospital, Dalian Medical University, Dalian, China; ^5^ College of Pharmacy, Dalian Medical University, Dalian, China; ^6^ Department of Clinical Pharmacology, College of Pharmacy, Dalian Medical University, Dalian, China

**Keywords:** cisplatin, nephrotoxicity, quercetin, polymeric micelles, antitumor potency

## Abstract

**Introduction:**

Cisplatin (DDP), a platinum-based chemotherapy drug, shows broad antineoplastic activity, however, its clinical use is limited by dose-dependent nephrotoxicity, a major challenge in cancer therapy. The purpose of this study was to investigate the mechanism by which quercetin-polyethylene glycol-polycaprolactone (Que-PEG-PCL) micelles simultaneously enhance the cytotoxicity of DDP against cancer cells and reduce its nephrotoxicity.

**Methods:**

Rodent models and HEK293 cells were used to evaluate the renoprotective effects of Que-PEG-PCL micelles. Pharmacokinetics focused on OCT2-mediated renal DDP disposition. Antitumor activity was assessed in CT26 cells and syngeneic tumors. Key assessments included oxidative stress, apoptosis, renal markers, and histopathology.

**Results:**

Que-PEG-PCL reduced DDP-induced nephrotoxicity, lowering creatinine and BUN to 42% and 38%. It also reduced oxidative stress and improved antioxidant activity. DDP plasma exposure increased to 323%, with renal clearance reduced to 14%, due to OCT2 inhibition. In a CT26 syngeneic model, combination therapy inhibited tumor volume by 84% compared to control group.

**Discussion:**

Que-PEG-PCL enhanced DDP’s therapeutic window by limiting renal accumulation and promoting tumor cell apoptosis. This dual-action strategy provides a novel approach for improving the clinical efficacy of DDP-based cancer therapy.

## 1 Introduction

DDP, an antineoplastic agent with broad-spectrum efficacy, was granted regulatory approval for oncological use in 1978, specifically for the management of ovarian and testicular malignancies (Conte et al., 2020). Despite its clinical benefits, DDP is commonly associated with nephrotoxicity, affecting about 30% of patients (Holditch et al., 2019). The nephrotoxic effects of DDP are intrinsically linked to its renal accumulation, mediated primarily by the organic cation transporter 2 (OCT2) in proximal tubular epithelial cells. This accumulation triggers a cascade of cellular insults, including oxidative stress, apoptosis, and inflammatory responses, ultimately leading to acute kidney injury (Fang et al., 2021). Consequently, strategies to mitigate DDP-induced nephrotoxicity without compromising its antitumor efficacy remain a critical unmet need in oncology.

The molecular mechanisms underlying DDP nephrotoxicity are multifaceted (Sprowl et al., 2014). Beyond OCT2-mediated uptake, DDP induces mitochondrial dysfunction, DNA damage, and activation of pro-apoptotic pathways, further exacerbating renal injury (Schoeberl et al., 2022; Ciarimboli, 2014). Pharmacological inhibition of OCT2 provides a promising strategy to limit renal DDP accumulation, however, finding safe, effective inhibitors that preserve antitumor activity remains a challenge.

Quercetin (Que), a flavonoid with a broad spectrum of beneficial effects, is known for its ability to inhibit reactive oxygen species (ROS), platelet activation, and apoptosis, as well as for providing endothelial protection, modulating inflammation, suppressing tumors, and offering renal protection (Olas and Bryś, 2020). A study of Que and DDP combination treatment revealed renoprotection via modulating key pathophysiological mechanisms and signaling pathways, without compromising DDP’s antitumor efficacy (Sanchez-Gonzalez et al., 2011). Notably, the research indicated that Que is a potent inhibitor of OCT2, as evidenced by studies on its interaction with eight flavonoids (Tan et al., 2023). Despite these advantages, Que’s clinical translation is hindered by poor aqueous solubility (<0.1 mg/mL), low oral bioavailability (<5%), and rapid metabolic clearance (Olas and Bryś, 2020). Additionally, Que undergoes significant enzymatic degradation in the gastrointestinal tract and exhibits rapid systemic clearance (Dabeek and Marra, 2019). To overcome these limitations, advanced drug delivery systems are urgently needed to enhance Que’s stability, bioavailability, and targeted delivery.

To enhance the bioavailability of Que, researchers have proposed a Que polymer micelle delivery system (Huang et al., 2022), which investigated nanosystems constructed from hydrophilic polyethylene glycol (PEG) conjugated with cationic polyethyleneimine (PEI) that encapsulated Que, achieving an hydrodynamic nanomedicine diameter of approximately 21 nm. Subsequent systemic applications using a mouse model of acute kidney injury demonstrated that Que-PEG-PEI effectively modulated key biomarker levels in serum. The extended retention of polymer micelles in clearance organs and their membrane affinity’s impact on drug transporters have gained interest. A recent study (Zhang Y. et al., 2021) indicated that the polymeric micelle carrier material can influence the function of organic cation transporters (OCTs) in healthy tissues. They found that mPEG2k-PCLx inhibited OCTs in a manner dependent on both concentration and the hydrophobic segment structure; notably, this inhibition decreased as the PCL segment length increased. Furthermore, PEG-PCL, a widely used block polymer, is known for its high transport capacity and favorable release profile. PEG–PCL nanocarriers offer prolonged circulation, improved drug stability, and high encapsulation efficiency, with minimal toxicity (Geszke-Moritz and Moritz, 2024). Given these advantages, the encapsulation of Que using PEG-PCL is expected to promote self-assembly into polymeric micelles in aqueous milieu, enabling the exploration nanodrug pharmacokinetics and interactions with DDP, ultimately enhancing therapeutic outcomes.

Building on this premise, the present study aimed to develop Que-PEG-PCL micelles and evaluate their dual functionality: (1) attenuating DDP-induced nephrotoxicity via OCT2 inhibition and antioxidative pathways, and (2) enhancing the antitumor efficacy of DDP by potentiating reactive oxygen species (ROS)-dependent apoptosis. We conducted a comprehensive investigation of the pharmacokinetic, pharmacodynamic, and mechanistic interactions between Que-PEG-PCL micelles and DDP, employing both *in vitro* models (HEK293 and OCT2-transfected cells) and *in vivo* rodent models of acute kidney injury and colorectal cancer. Our findings provide a novel nanotherapeutic strategy to expand the therapeutic window of DDP by concurrently mitigating its renal toxicity and augmenting its antineoplastic potency. To the best of our knowledge, there has been a lack of studies reporting the synergistic interactions between Que-PEG-PCL micelles and cisplatin in enhancing therapeutic efficacy while concurrently reducing associated toxicities.

## 2 Materials and methods

### 2.1 Reagents and chemicals

Monomethoxy polyethylene glycol (MPEG, M_w_ = 2,000 Da) was purchased from Ponsure Biotechnology Co., Ltd. (Shanghai, China). ε-caprolactone and stannous octoate were obtained from Sigma–Aldrich (Shanghai, China). DDP was purchased from Shanghai Yuanye Biology Technology Co., Ltd. (Shanghai, China). Que and Sodium diethyldithiocarbamate (DDTC) were sourced from Dalian Meilun Biology Technology Co., Ltd. (Dalian, China). Palladium (Pd) was purchased from Shanghai Aladdin Biochemical Technology Co., Ltd. (Shanghai, China) with a certified purity of ≥99.9% (w/w). The CCK-8 assay kit was procured from Yunhe Biotechnology Co., Ltd. (Nanjing, China). The platinum standard solution was purchased from Shanghai Aladdin Biochemical Technology Co., Ltd. (Shanghai, China). Dichlorodiamminepalladium was supplied by Bide Pharmatech Co., Ltd. (Shanghai, China), ethyl acetate from Tianjin Kermel Chemical Reagent Co., Ltd. (Tianjin, China), and sodium hydroxide from Tianjin Continental Chemical Reagent Factory (Tianjin, China). Disodium ethylenediaminetetraacetate (EDTA-Na_2_) and BCA Protein Assay Kit were provided by Beijing Solarbio Science & Technology Co., Ltd. (Beijing, China). Kits for the quantification of superoxide dismutase (SOD), creatinine (Cr), malondialdehyde (MDA), and blood urea nitrogen (BUN), were obtained from Nanjing Jiancheng Bioengineering Institute (Nanjing, China). Fetal bovine serum (FBS) was purchased from Thermo Fisher Scientific (Shanghai, China). Murine CT26 colon cancer cell line was kindly provided by Prof. Likun Gong (Shanghai Institute of Materia Medica, China). The HEK293 and OCT2-HEK293 cells were kindly provided by Prof. Yuichi Sugiyama (Sugiyama Laboratory, RIKEN, Japan).

### 2.2 Experimental animals and cell culture

Male Wistar rats (200 ± 20 g) and male BALB/c mice (20 ± 2 g) were purchased from Liaoning Changsheng Biotechnology Co., Ltd. (Benxi, China). Human embryonic kidney (HEK293) cells and hOCT2-transfected HEK293 cells were propagated in Dulbecco’s Modified Eagle Medium (DMEM) containing 10% FBS. Mouse colon cancer CT26 cells were grown in Roswell Park Memorial Institute (RPMI-1640) medium. All cell cultures were maintained at 37°C in a humidified atmosphere composed of 5% CO_2_ and 95% O_2_. The rats and mice were housed under sterile conditions at an ambient temperature of approximately 25°C and a relative humidity ranging from 30% to 70%, with free access to standard laboratory diet and water. All experimental animals, protocols, and procedures were approved by the Dalian Medical University’s Laboratory Animal Ethics Committee (approval number: AEE24046).

### 2.3 Synthesis of PEG-PCL

PEG-PCL was synthesized from monomer of ε-caprolactone and the macroinitiator of MeO-PEG-OH (*M*_w_: 2,000 Da) in the presence of stannous octoate as a catalyst via ring opening polymerization. Briefly, weighted amounts of ε-caprolactone, MeO-PEG-OH and 0.05% stannous octoate were reacted at 140°C for 24 h in a sealed glass ampoule. All synthesis was carried out under an oxygen- and moisture-free environment. After polymerization, the reacted product was dissolved in DCM, followed by precipitation in excess cold diethyl ether to obtain the crude product. The solid product was obtained by filtration and vacuum dried for 48 h.

### 2.4 Characterization of PEG-PCL

The chemical structure of PEG-PCL copolymer were characterized by ^1^HNMR in deuterated CDCl_3_ at 600 Hz (Bruker Avance Neo 600, Germany). The molecular weight and molecular weight distribution of PEG-PCL copolymer were measured by gel permeation chromatography (GPC) (LC-20ADXR, Shimadzu, Japan) equipped with a RID-20 A refractive index detector. The GPC analysis was conducted at 35°C, with tetrahydrofuran (THF) as the eluent and a flow rate of 1 mL/min. The calibration was performed using polystyrene standards.

### 2.5 Preparation of Que-loaded micelles

Que-PEG-PCL micelles were prepared by a thin film hydration method. The Que and PEG-PCL (mass ratio: 1:30) were dissolved in 30 mL of acetonitrile. The resulting solution was evaporated to form a thin film, followed by drying under vacuum overnight to ensure complete removal of residue organic solvent. Subsequently, the dried film was hydrated with pre-heated PBS (pH 7.4) for 3 min to facilitate complete hydration. The hydrated solution was then filtered through a 0.22 μm syringe filter to remove any undissolved or precipitated drug particles. The empty PEG-PCL micelles were prepared following the same procedure, but without Que.

### 2.6 Characterization of Que-loaded micelles

The Que content within the Que-PEG-PCL micelles was measured via HPLC (Shimadzu, LC-16, Japan) a Diamonsil C_18_ column. The mobile phase was methanol/0.05% aqueous phosphoric acid (65:35, v/v) with the flow rate of 1 mL/min. The column temperature was maintained at 25°C and the UV detector wavelength was 370 nm. The drug loading (DL%) and encapsulation efficiency (EE%) were calculated according to Equations 1, 2:

$$DL\left(\%\right)=\frac{Weight\;of\;drug\;in\;micelles}{Total\;weight\;of\;the\;drug\;and\;polymer}\times 100$$ (1)

$$EE\left(\%\right)=\frac{Weight\;of\;the\;drug\;in\;micelles}{Weight\;of\;the\;feeding\;drug}\times 100$$ (2)

Zeta potential, particle size and size distribution of the micelles were determined using dynamic light scattering detector (Zetasizer Nano ZS90, Malvern, United Kingdom). The morphologies and structures of Que-loaded micelles, as well as the corresponding plain micelles, were examined after staining with phosphotungstic acid (2 wt%) using a transmission electron microscope (Jeol JEM-2000EX, Tokyo, Japan).

### 2.7 Induction of acute kidney injury by DDP in rats

The rats (n = 3 for each group) were randomly assigned to six experimental cohorts: (1) Control; (2) PEG-PCL (75 mg/kg, i.v., single dose); (3) Que-PEG-PCL (2.5 mg/kg, i.v., single dose); (4) DDP (15 mg/kg, i.p., single dose); (5) DDP + PEG-PCL (DDP: 15 mg/kg, i. p., single dose; PEG-PCL: 75 mg/kg, i.v., single dose); (6) DDP + Que-PEG-PCL (DDP: 15 mg/kg, i.p., single dose; Que-PEG-PCL: 2.5 mg/kg, i.v., single dose). DDP was administered intraperitoneally 2 h after the final injection on day 5 for groups 4, 5, and 6. After 8 days of DDP injections, the rats were subjected to anesthesia via intraperitoneal administration of pentobarbital sodium (20 mg/kg). Blood samples were collected from the abdominal aorta post-anesthesia induction with sodium pentobarbital. The plasma were then obtained by centrifuging at 4,500 rpm for 15 min, and then stored at −80°C for later analysis. Kidneys were excised, weighed, and fixed in 4% paraformaldehyde for further histological examinations. Biochemical parameters (Cr, BUN, SOD and MDA) were quantified according to the manufacturer’s protocols.

### 2.8 Renal TUNEL staining

Paraffin-embedded kidneys were sectioned at a thickness of 5 μm. Sections were subjected to the subsequent TUNEL-staining using an apoptosis detection kit (Roche Diagnostics) to identify apoptotic cells. Following TUNEL staining, the sections were counterstained with DAPI to visualize nuclei. The stained slides were sealed and examined under fluorescence microscopy. The TUNEL staining fluorescence was quantitatively analyzed using ImageJ to calculate the apoptotic index (ratio of fluorescent-positive cells to the total cells). Renal tubular necrosis: using a semiquantitative scoring system from 0 to 4 (0: no injury; 1: ≤25% tubular injury; 2: 25%–50%; 3: 50%–75%; 4: ≥75% or extensive necrosis).

### 2.9 Cytotoxicity

HEK293 and hOCT2-HEK293 cells were seeded into 96-well plates (8 × 10^3^ cells/well) and treated with DDP (0.1–500 μM) for 24 h. Cell viability was assessed using the CCK-8 assay, according to the manufacturer’s instructions. For treatments with PEG-PCL and QUE-PEG-PCL, the gradient concentrations employed were 15, 30, 75, 150, 300, 450, and 600 μg/mL for PEG-PCL, and 0.5, 1, 2.5, 5, 10, 15, and 20 μg/mL for QUE-PEG-PCL, respectively.

### 2.10 Quantification of apoptotic cells

HEK293 cells seeded into 6-well plates were treated with 45 μM DDP alone, or in combination with 600 μg/mL PEG-PCL or Que-PEG-PCL (20 μg/mL Que) for 24 h. Cells were then trypsinized, centrifuged, and resuspended in binding buffer. Subsequently, Annexin V-FITC and propidium iodide (PI) (2:1 M ratio) were added to stain the cells for 15 min. Thereafter, stained cells were analyzed by flow cytometry (Agilent NovoCyte Advanteon).

### 2.11 Pharmacokinetic investigations

Healthy male Wistar rats (n = 3) were randomly allocated to one of four experimental cohorts: (1) the DDP (0.5 mg/kg, intravenous, single bolus) group; (2) the DDP (0.5 mg/kg, intravenous, single bolus) + PEG-PCL (75 mg/kg, intravenous, single bolus) group; (3) the DDP (0.5 mg/kg, intravenous, single bolus) + Que-PEG-PCL (2.5 mg/kg, intravenous, single bolus) group; (4) the DDP (0.5 mg/kg, intravenous, single bolus) + Quinidine (6 mg/kg, intravenous, single bolus) group. The administration of DDP preceded that of PEG-PCL or Que-PEG-PCL by 0.5 h. Venous blood samples were collected from the jugular vein at predetermined time points (0.5, 1, 2, 4, 6, 8 and 12 h) following DDP administration (Diehl et al., 2001). An equivalent volume of saline solution was concurrently administered. Urine was collected via cystostomy at 1 h, 2 h, 3 h, 4 h, 6 h, 8 h, and 12 h post-DDP administration.

### 2.12 HPLC-MS/MS analysis of DDP

A 10% (w/v) sodium diethyldithiocarbamate (DDTC) solution was freshly prepared in 0.1 mol/L NaOH. For derivatization, 20 μL internal standard (palladium (Pd)) and 20 μL DDTC were added to 30 μL biosamples and reacted for 1 min. Then, 200 μL ethyl acetate was added, mixed gently, and centrifuged (11,180 × g, 1 min, 20°C) using Thermo Scientific™ Sorvall™ Legend™ Micro 21 R (Thermo Fisher Scientific, Waltham, MA, United States) to extract DDP-DDTC. A 150 μL supernatant was dried under nitrogen and reconstituted in 100 μL mobile phase. The prepared samples were analyzed using with an API 3200 triple quadrupole mass spectrometer (Applied Biosystems, United States) coupled with Agilent HP1200 liquid chromatography system (Agilent Technologies, United States) (Shaik et al., 2017).

### 2.13 Cellular uptake analysis in OCT2-transfected cells

hOCT2-HEK293 and mock-HEK293 cells were seeded in 24-well plates and cultured overnight. PEG-PCL or Que-PEG-PCL was added (Que: 0.5–20 μg/mL; PEG-PCL: 15–600 μg/mL) and incubated for 30 min. Cells were then treated with DDP (500 μM) for another 30 min, followed by lysis through three freeze–thaw cycles in deionized water at −80°C. The lysates were collected in deionized water for DDP analysis.

### 2.14 Intracellular reactive oxygen species (ROS) assessment

CT26 cells in 24-well plates were treated for 8 h with 50 μM DDP alone or combined with PEG-PCL (600 μg/mL), Que (20 μg/mL), or Que-PEG-PCL (Que: 1, 10, or 20 μg/mL). After rinsing with PBS, cells were stained with 10 μM DCFH-DA for 30 min, trypsinized, and analyzed for fluorescence (excitation: 488 nm; emission: 525 nm) using a multifunctional microplate reader (Tecan, Switzerland). Protein concentration was quantified by BCA assay.

### 2.15 Measurement of mitochondrial membrane potential

CT26 cells were treated with DDP alone or in combination with PEG-PCL, free Que, or Que-PEG-PCL for 8 h, as previously described. Cells were then trypsinized and resuspended with 200 μL JC-1 staining solution for 20 min at 37°C. After two washes with 1× JC-1 buffer, green (Ex: 490 nm, Em: 530 nm) and red (Ex: 525 nm, Em: 590 nm) fluorescence were measured using a multifunctional microplate reader.

### 2.16 CT26 cells syngeneic murine model

Male BALB/c mice were inoculated subcutaneously with CT26 cells (5 × 10^5^ cells/mouse). Once the mean tumor volume reached 50–100 mm^3^, The mice were randomly divided into seven groups (n = 5): (1) control; (2) DDP (2 mg/kg); (3) DDP (2 mg/kg) combined with PEG-PCL (120 mg/kg); (4) DDP (2 mg/kg) combined with Que (4 mg/kg); (5) DDP (2 mg/kg) combined with Que-PEG-PCL (1 mg/kg Que) (1); (6) DDP (2 mg/kg) combined with Que-PEG-PCL (2 mg/kg Que) (2); and (7) DDP (2 mg/kg) combined with Que-PEG-PCL (4 mg/kg Que) (3). Starting on day 3 post-inoculation, DDP was administered intravenously for four consecutive days. PEG-PCL, Que (4 mg/kg) and Que-PEG-PCL was administered intravenously administered once daily for seven consecutive days. Body weight and tumor volume were measured daily throughout the experimental period. Serum biochemical parameters (Cr, BUN, SOD, MDA) were analyzed determined according to the manufacturer’s instructions. Major organs were subjected to t histopathological assessment via hematoxylin and eosin (H&E) staining.

### 2.17 Statistical analysis

GraphPad Prism 7.0 software (GraphPad Software Inc., San Diego, CA, RRID: SCR_002798) was used to handle the statistical analyses. All results were obtained from at least three independent experiments. The statistical analysis was performed using one-way ANOVA followed by Tukey’s *post hoc* test when comparing multiple independent groups. For comparisons between two different groups, the unpaired Student’s t-test was employed. The results are delineated as the mean ± standard deviation (S.D.). The threshold for determining statistical significance was set at a p-value less than 0.05.

## 3 Results

### 3.1 Synthesis and characterizations of PEG-PCL

**FIGURE 1:**
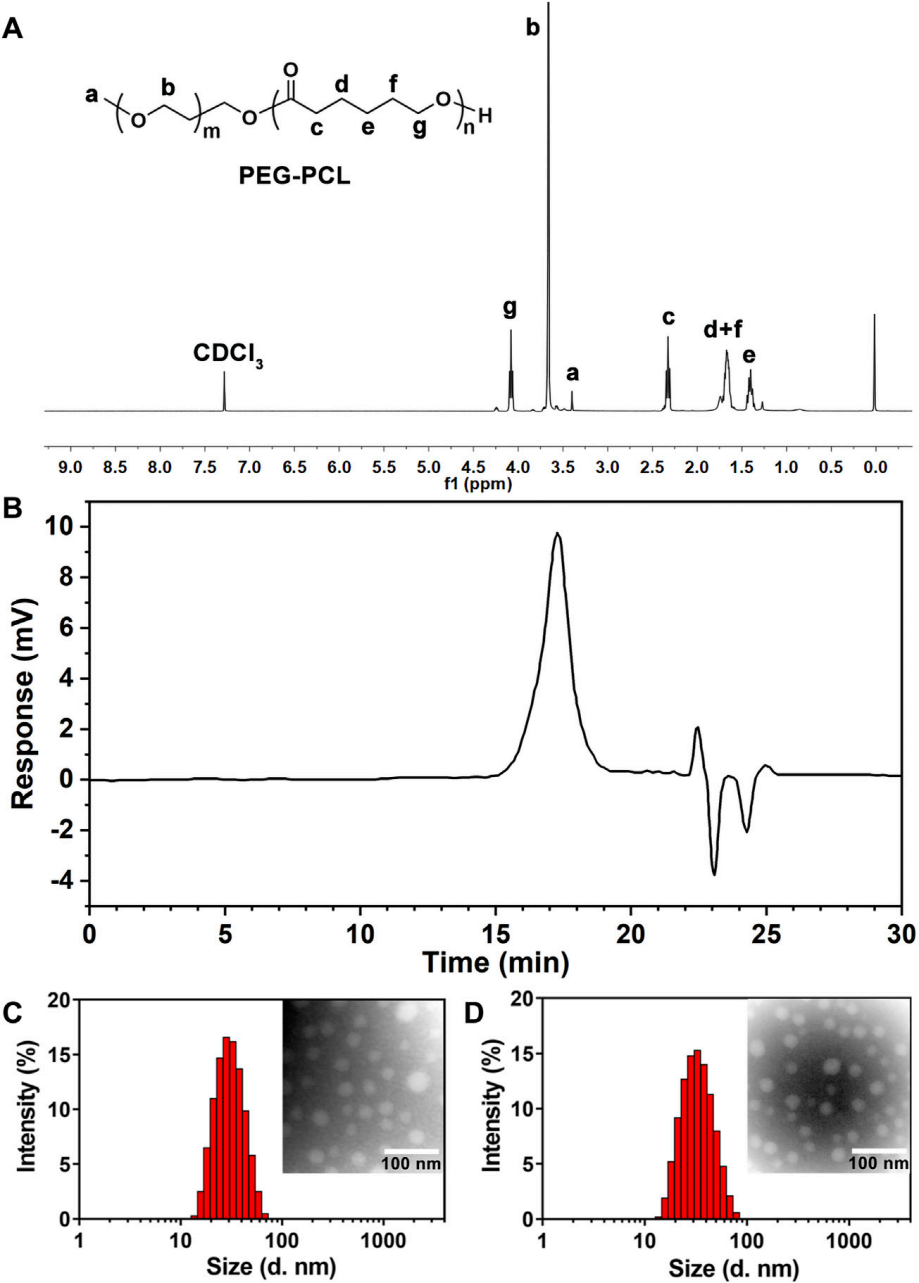
**(A)** ^1^HNMR spectrum of block polymer PEG-PCL. **(B)** The typical GPC spectrum of PEG-PCL block copolymer. **(C)** Size distribution and TEM morphology of PEG-PCL (×200000). **(D)** Size distribution and TEM morphology of Que-PEG-PCL (×200000).

PEG-PCL was synthesized via ring-opening polymerization of ε-caprolactone and MPEG 2000 using stannous octoate as a catalyst (Supplementary Figure S1). The chemical structure of the block copolymeric PEG-PCL was unequivocally confirmed by ^1^HNMR spectroscopy (Figure 1A). The characteristic resonances of both PEG (δ_a_ = 3.40 ppm and δ_b_ = 3.66 ppm) and PCL (δ_c_ = 2.33 ppm, δ_d,f_ = 1.67 ppm, δ_e_ = 1.38 ppm, δ_g_ = 4.08 ppm) were identified, suggesting successful copolymerization. Further validation of the polymer’s molecular weight distribution was achieved through gel permeation chromatography (GPC). The chromatogram (Figure 1B) revealed a symmetrical unimodal peak with a weight-average molecular weight (*M*_w_) of approximately 4,578 Da and a polydispersity index (PDI) of 1.16 (Supplementary Table S1), demonstrating a well-controlled synthesis with low heterogeneity. These structural insights collectively affirmed the precision of the synthetic methodology and the suitability of PEG-PCL for subsequent micellar formulations.

### 3.2 Characterization of Que-loaded micelles

Que-encapsulated PEG-PCL micelles were prepared via a thin-film hydration method. High-performance liquid chromatography (HPLC) revealed an encapsulation efficiency (EE) of 86.77% ± 2.34% and a drug loading (DL) of 2.80% ± 0.80% for Que-PEG-PCL micelles (Supplementary Table S2). Dynamic light scattering (DLS) measurements showed a hydrodynamic diameter of 29.23 ± 3.43 nm and a near-neutral zeta potential of −0.08 ± 0.10 mV (Supplementary Table S3). Transmission electron microscopy (TEM) revealed that both blank and Que-loaded micelles exhibited a well-defined spherical morphology with uniform size and homogeneous dispersion (Figures 1C,D).

### 3.3 Renoprotective effects of Que-PEG-PCL against DDP-induced nephrotoxicity

**FIGURE 2:**
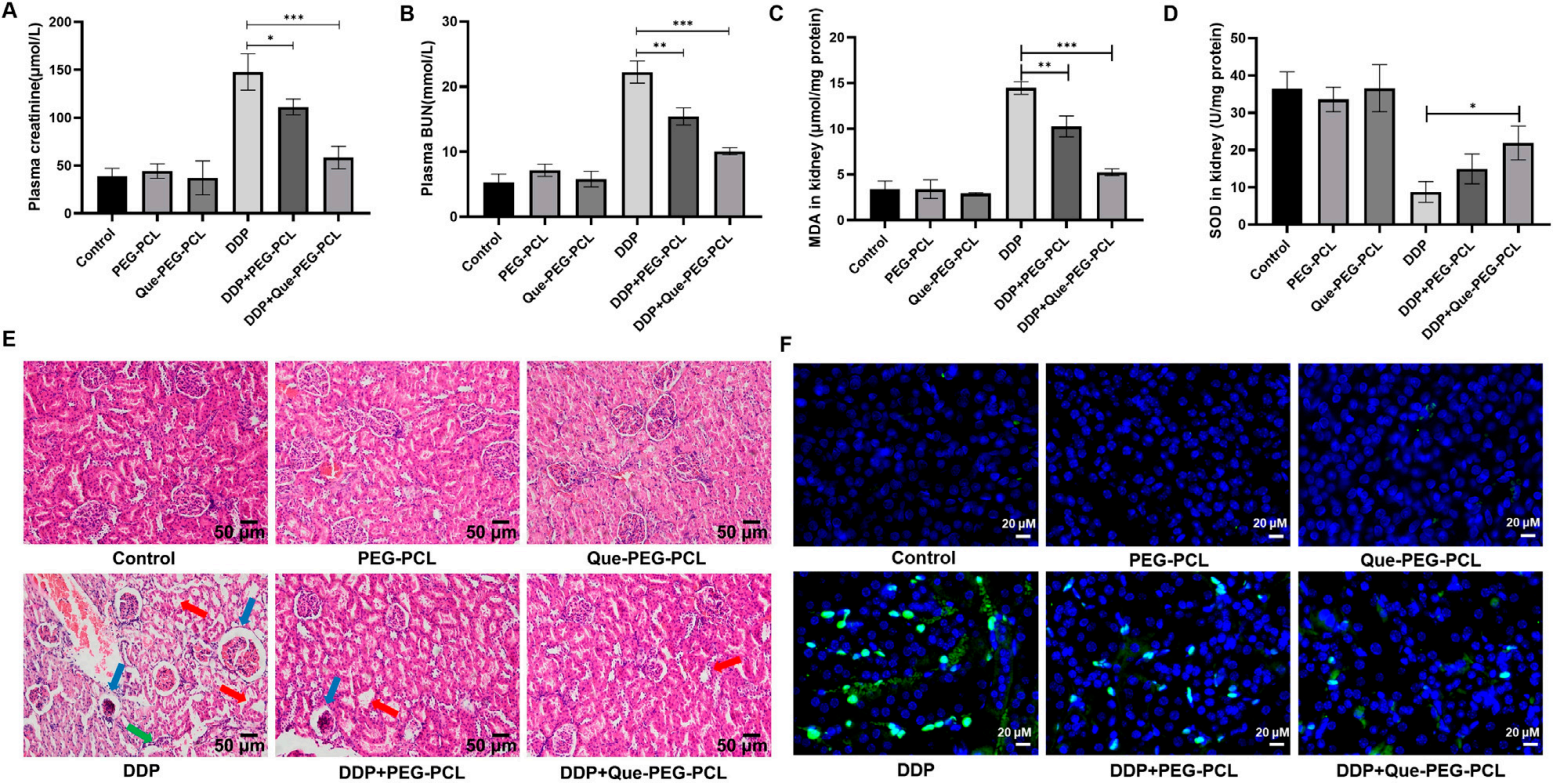
Que-PEG-PCL alleviated DDP-induced nephrotoxicity, oxidative stress, and apoptosis in rats. **(A)** Serum creatinine (Cr), **(B)** blood urea nitrogen (BUN), **(C)** malondialdehyde (MDA, oxidative stress marker), **(D)** superoxide dismutase (SOD, antioxidant enzyme activity). **(E)** Hematoxylin and eosin (H&E) staining of renal tissues (scale bar: 50 μm). **(F)** Terminal deoxynucleotidyl transferase dUTP nick end labeling (TUNEL) staining for apoptosis (green: TUNEL-positive cells). Statistical significance is indicated as *p < 0.05, **p < 0.01, ***p < 0.001 compared to the DDP group. Data are presented as mean ± S.D., with n = 3.

Compared to controls, PEG-PCL and Que-PEG-PCL treatments did not significantly affect renal function, indicating no associated nephrotoxicity (Figures 2A,B). In contrast, DDP monotherapy led to marked body weight loss and significant increases in renal indices, serum creatinine (Cr), and blood urea nitrogen (BUN), indicating renal impairment (Figures 2A,B). Co-administration of PEG-PCL with DDP significantly preserved body weight and reduced renal indices, serum Cr, and BUN levels, highlighting its renoprotective effects against DDP-induced renal injury (Figures 2A,B). Therapeutic intervention with Que-PEG-PCL further enhanced recovery from DDP-induced renal damage, indicating a stronger protective effect against nephrotoxicity (Figures 2A,B). Mouse kidneys exhibited obvious acute tubular necrosis, including vacuolization in the glomerular mass, tubular distention, cellular necrosis, and epithelial detachment. These DDP-induced renal pathological alterations were mitigated by PEG-PCL administration and further attenuated by Que-PEG-PCL, thereby substantiating the renoprotective efficacy of both PEG-PCL and Que-PEG-PCL against DDP-induced nephrotoxicity (Figure 2E). Mechanistic analysis revealed that DDP-induced nephrotoxicity was associated with elevated oxidative stress, as evidenced by increased MDA levels and decreased SOD activity (Figures 2C,D). The therapeutic intervention with PEG-PCL precipitated a marked reduction in MDA levels and a restoration of SOD activity relative to DDP monotherapy. Moreover, Que-PEG-PCL administration elicited an exacerbated effect, suggesting a synergistic antioxidant effect due to the amalgamation of PEG-PCL and Que, both of which are reputed for their antioxidant properties capable of mitigating oxidative stress (Figures 2C,D). The TUNEL assay, employed to quantify apoptotic cell death, revealed minimal green fluorescence in the renal tissues of the control, PEG-PCL, and Que-PEG-PCL groups, thereby corroborating their non-toxicity (Figure 2F). In stark contrast, the renal tissues of the DDP group exhibited a substantial number of TUNEL-positive cells, indicative of pronounced apoptosis (Figure 2F). Both PEG-PCL and Que-PEG-PCL treatments induced a progressive reduction in the number of TUNEL-positive cells, with Que-PEG-PCL demonstrating a more pronounced effect, thereby highlighting its efficacy in inhibiting DDP-induced apoptosis within rat kidneys (Figure 2F).

### 3.4 Cytoprotective, antioxidant, and anti-apoptotic effects of Que-PEG-PCL in DDP-treated HEK293 cells

**FIGURE 3:**
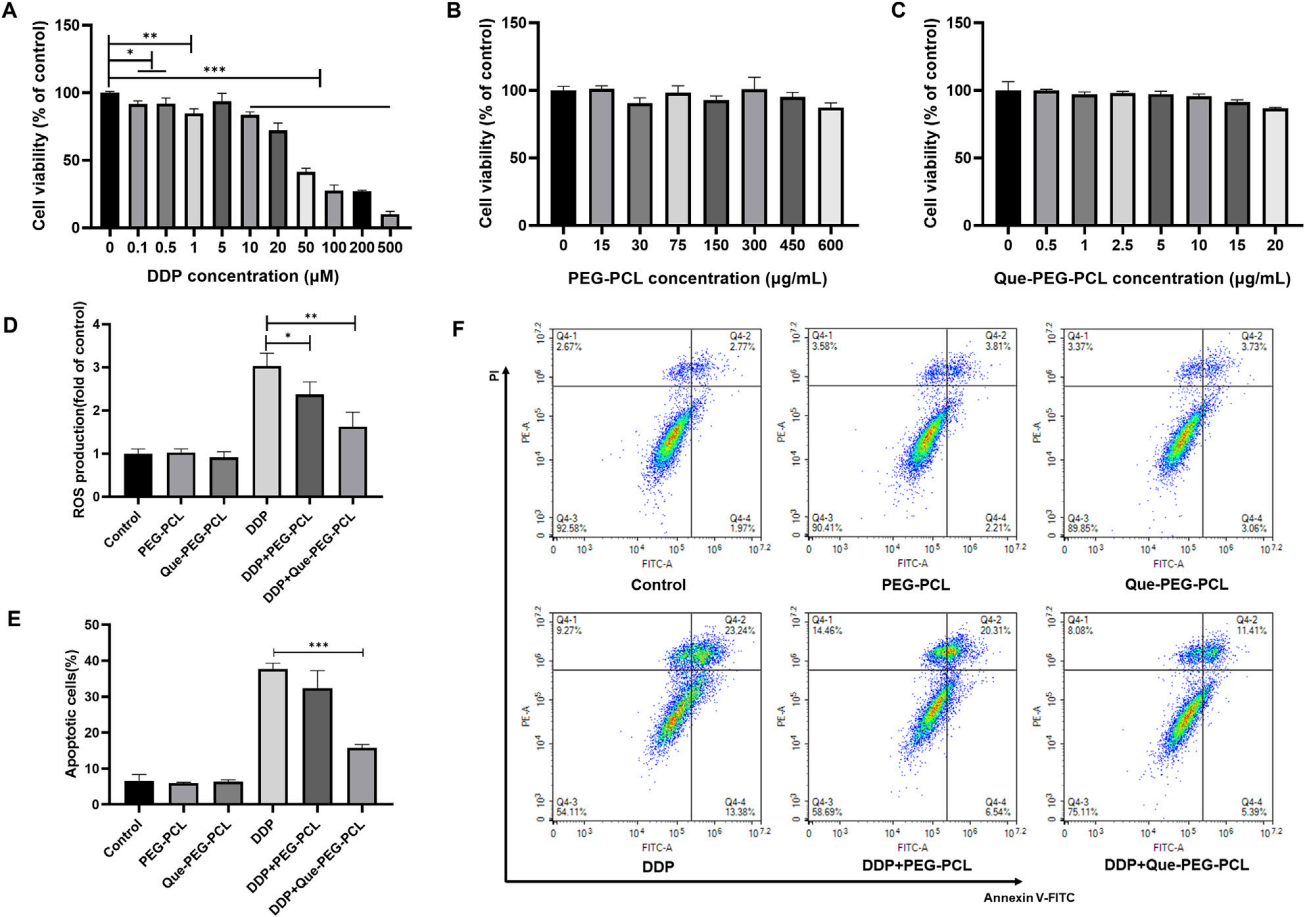
Que-PEG-PCL mitigates DDP-induced ROS production and apoptosis in HEK293 cells. **(A)** Impact of DDP on the viability of HEK293 cells. **(B)** Influence of PEG-PCL on the viability of HEK293 cells. **(C)** Effect of Que-PEG-PCL on the viability of HEK293 cells. **(D)** Alterations in ROS levels following incubation with PEG-PCL, Que-PEG-PCL, and DDP individually or in combination. **(E,F)** Influence of PEG-PCL and Que-PEG-PCL on DDP-induced apoptosis in HEK293 cells. Statistical significance is denoted as *p < 0.05, **p < 0.01, ***p < 0.001 compared to the DDP group. Data are presented as mean ± S.D., with n = 3.

**FIGURE 4:**
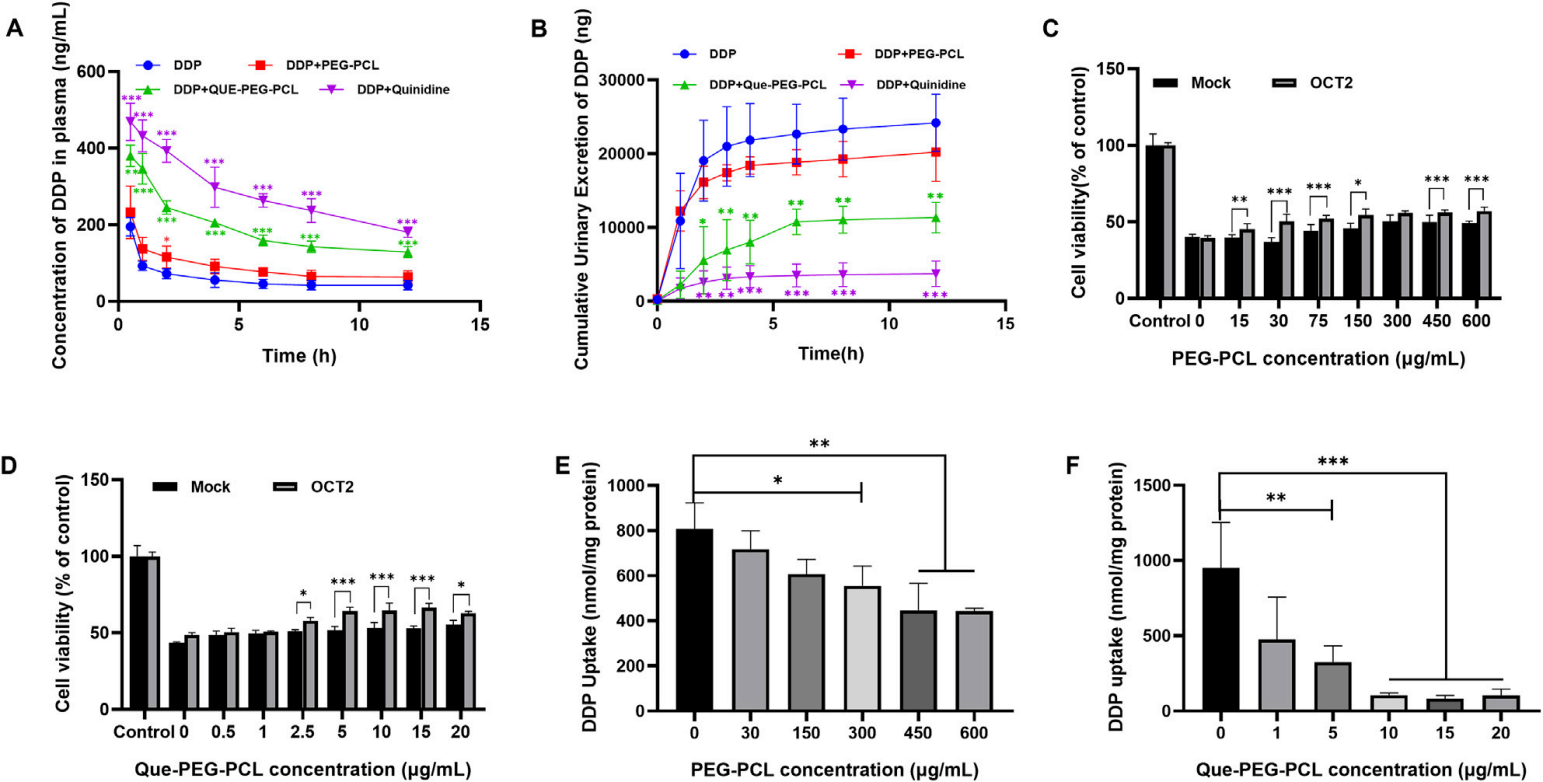
Effects of Que-PEG-PCL on the pharmacokinetics of DDP in rats and effects of OCT2 and DDP on the cytotoxicity and intracellular accumulation of PEG-PCL and Que-PEG-PCL in HEK293 cells and hOCT2-HEK293 cells. **(A)** Impact of PEG-PCL, Que-PEG-PCL, and Quinidine on the plasma concentration of DDP in rats. **(B)** Influence of PEG-PCL, Que-PEG-PCL, and Quinidine on the urinary excretion of DDP in rats. The cytotoxicity of PEG-PCL (15–600 μg/mL) **(C)** and Que-PEG-PCL **(D)** (0.5–20 μg/mL) in mock- and hOCT2-HEK293 cells. **(E,F)** Concentration-dependent inhibition curve of PEG-PCL and Que-PEG-PCL on DDP uptake in hOCT2-HEK293 cells. Statistical significance is denoted as *p < 0.05, **p < 0.01, ***p < 0.001 compared to the DDP group. #p < 0.05, #p < 0.01, #p < 0.001 vs. mock cells. Data are presented as mean ± standard deviation (S.D.), with n = 3.

The assessment of the biosafety and cytoprotective mechanisms of the micellar formulations against DDP-induced cytotoxicity was further conducted *in vitro* utilizing HEK 293 cell line as a model system. DDP induced a dose-dependent decrement in cellular viability, with the IC_50_ value ascertained to be 45.28 μM (Figure 3A; Supplementary Figure S2A). In contrast, PEG-PCL and Que-PEG-PCL exhibited minimal cytotoxicity across concentrations of 15–600 μg/mL, maintaining cellular viability above 80% (Figures 3B,C). The PEG-PCL and Que-PEG-PCL formulations demonstrated a dose-dependent amelioration of DDP-induced cytotoxicity, with Que-PEG-PCL exhibiting higher enhanced cytoprotective properties (Figures 4C,D). These results suggest that PEG-PCL possesses intrinsic cytoprotective attributes, and the conjugation of Que in Que-PEG-PCL augments this effect, potentially through synergistic interactions between PEG-PCL and Que. The cytoprotective capacity of Que-PEG-PCL against DDP-induced cytotoxicity was delineated through quantification of ROS production and apoptotic cell death in HEK293 cells. DDP treatment was associated with a significant upregulation of ROS levels in comparison to the control cohort, while the intervention with PEG-PCL and Que-PEG-PCL resulted in a dose-dependent diminution of ROS, with Que-PEG-PCL exhibiting a more pronounced suppressive effect (Figure 3D). Flow cytometry suggested no significant increase in apoptosis in PEG-PCL or Que-PEG-PCL treated groups compared to controls, whereas the DDP group exhibited a significant rise in apoptosis (Figures 3E,F). Co-administration of DDP with PEG-PCL or Que-PEG-PCL reduced apoptosis, with Que-PEG-PCL showing a more pronounced effect, confirming its antioxidant and anti-apoptotic properties in mitigating DDP-induced cytotoxicity.

### 3.5 Modulation of DDP pharmacokinetics and renal excretion by Que-PEG-PCL

A pharmacokinetic analysis assessed the effect of Que-PEG-PCL on DDP’s systemic exposure and urinary elimination, revealing its nephroprotective mechanisms. Concurrent administration of PEG-PCL, Que-PEG-PCL, or quinidine significantly increased the plasma concentration of DDP. Furthermore, the half-life (t_1/2_) and the plasma concentration-time curve (AUC) of DDP in the coadministration groups were increased, and the plasma clearance (CL_p_) of DDP was decreased (Table 1; Figure 4A). Que-PEG-PCL significantly decreased cumulative urinary excretions and CL_r_ of DDP compared to the DDP alone group (Table 1; Figure 4B). The inclusion of quinidine, a well-characterized inhibitor of OCT2, provided evidence that the observed modulations in DDP’s CL_r_ may be attributed to the inhibition of OCT2-mediated transport. To further clarify the role of OCTs in the pharmacokinetic interaction between micelles and DDP, cytotoxicity and uptake of DDP were determined using hOCT2-HEK293 cells. In the hOCT2-HEK293 cells, combined use of PEG-PCL and Que-PEG-PCL increased cell survival by 17.6% and 19.3%, respectively (Figures 4C,D). In a similar experiment, in mock-HEK293 cells, the cell survival rate only increased by only 9.1% and 11.9%, respectively, with the combination of PEG-PCL and Que-PEG-PCL (Figures 4C,D). The results indicated that in addition to the inherent cell protective activity of the material itself, PEG-PCL and Que-PEG-PCL also provide additional protection via inhibiting OCT2 (Figures 4C,D). In the Que-PEG-PCL group, the survival rates of both mock- and hOCT2-HEK293 cells increased (Figure 4D). These results underscore the potential of Que-PEG-PCL to confer protection against DDP-induced cytotoxicity in hOCT2-HEK293 cells, possibly through the modulation of OCT2-mediated DDP uptake. Cell uptake experiments revealed that both PEG-PCL and Que-PEG-PCL inhibited DDP uptake in a concentration-dependent manner, with Que-PEG-PCL showing a significantly stronger effect. This supports prior data suggesting that Que-PEG-PCL inhibits OCT2-mediated DDP uptake, potentially reducing its nephrotoxic effects (Figures 4E,F).

**TABLE 1:** Pharmacokinetic parameters of DDP after intravenous administration of DDP with or without PEG-PCL, Que-PEG-PCL or Quinidine in rats.

Parameter	DDP	DDP + PEG-PCL	DDP + Que-PEG-PCL	DDP + quinidine
AUC _(0–12)_ (μg/L·h)	784.9 ± 10.3	1,346.1 ± 138.5^*^	2,532.1 ± 132.2^***^	3,571.5 ± 358.1^***^
t_1/2_ (h)	1.56 ± 0.05	2.48 ± 0.69	3.53 ± 0.82	9.87 ± 1.39^***^
CL_p_ (L/h/kg)	0.38 ± 0.04	0.21 ± 0.01^**^	0.12 ± 0.004^**^	0.09 ± 0.009^***^
CL_r_ (L/h/kg)	0.15 ± 0.023	0.077 ± 0.02^***^	0.022 ± 0.003^***^	0.005 ± 0.002^***^

*p < 0.05, **p < 0.01, ***p < 0.001 vs. DDP only group. mean ± S.D., n = 3. AUC (0–12): Area under the plasma concentration-time curve from 0 to 12 h; t_1/2_: Elimination half-life; CL_p_: Plasma clearance; CL_r_: Renal clearance.

### 3.6 Synergistic antitumor effects of DDP and Que-PEG-PCL in a colorectal carcinoma mouse model

**FIGURE 5:**
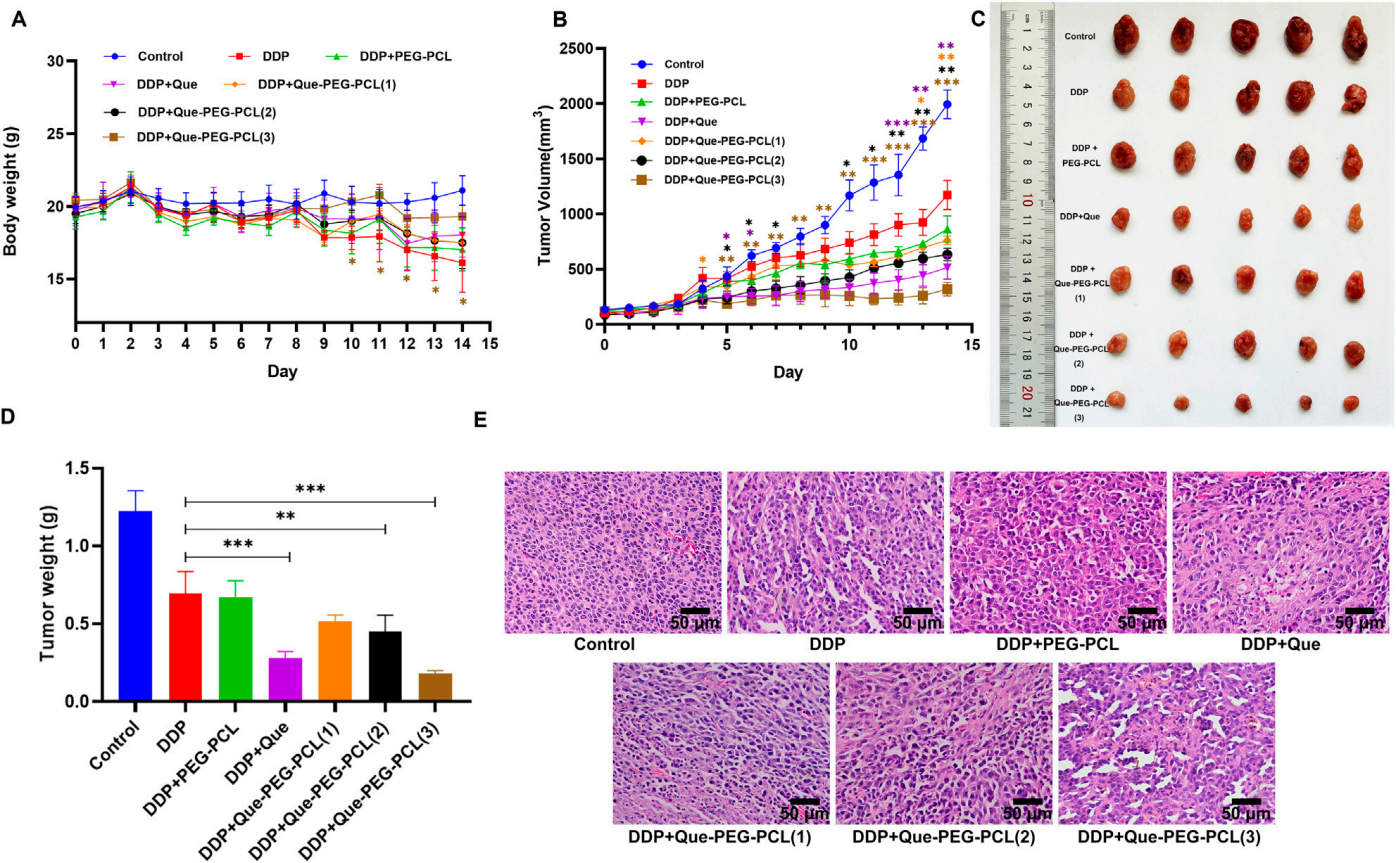
Influence of Que-PEG-PCL on the antitumor activity of DDP in mice with transplanted tumors. **(A)** Temporal changes in body weight of CT26 tumor-bearing mice following various therapeutic interventions. **(B)** Dynamics of tumor volume in CT26 tumor-bearing mice after different treatments. **(C)** Morphological alterations in tumors in CT26 tumor-bearing mice post-treatment. **(D)** Variations in tumor weight in CT26 tumor-bearing mice following diverse treatments. **(E)** Histopathological analysis of tumors in CT26 tumor-bearing mice treated with various formulations, as assessed by hematoxylin-eosin (HE) staining (scale bar: 50 μm). Statistical significance is indicated as *p < 0.05, **p < 0.01, ***p < 0.001 compared to the DDP group. Data are expressed as mean ± standard deviation (S.D.), with n = 3.

To evaluate the therapeutic modulatory effects of Que-PEG-PCL on DDP efficacy, mice engrafted with subcutaneous CT26 syngeneic tumors were treated with DDP, either as a monotherapy or in combination with PEG-PCL, Que, or Que-PEG-PCL. Body weight, a sensitive biomarker of systemic toxicity, was stringently monitored subsequent to DDP administration. Mice within the control group exhibited a progressive increase in body weight, in contradistinction to those treated with DDP, which demonstrated a precipitous decline, indicative of significant side effects associated with DDP. Compared with the DDP group, the body weight of Que-PEG-PCL groups showed a significant increase (Figure 5A). DDP treatment effectuated a significant suppression of tumor growth, with a 30% reduction in tumor volume (Figure 5B) and 60% reduction in weight relative to the control group (Figure 5A). The concomitant administration of Que or PEG-PCL resulted in a modest yet statistically significant potentiation of DDP’s antineoplastic activity in tumor-bearing mice. Notably, Que-PEG-PCL enhanced the antitumor effect of DDP in a dose-dependent manner, with the combination treatment (DDP + Que-PEG-PCL at 4 mg/kg) achieving a tumor inhibition rate of 84%, surpassing the efficacy of Que alone. This result suggests that Que-PEG-PCL micelles may enhance the antineoplastic effects of Que in colorectal cancer models, potentially due to the improved solubility and stability of Que, thereby elevating drug concentrations at the tumor site and amplifying its antineoplastic impact. Although complete tumor ablation was not achieved in the DDP and Que-PEG-PCL group, this treatment regimen demonstrated the most efficacious therapeutic outcomes among all regimens evaluated (Figures 5C,D). Histopathological examination revealed the presence of necrosis and nuclear pyknosis in the tumor tissue of the DDP group, with an increase in necrotic areas in a dose-dependent manner upon co-administration of Que-PEG-PCL (Figure 5E). Collectively, these findings suggest that the combinatorial treatment with Que-PEG-PCL and DDP exerts a synergistic effect to inhibit tumor growth.

### 3.7 Que-PEG-PCL synergism in DDP antineoplastic therapy

**FIGURE 6:**
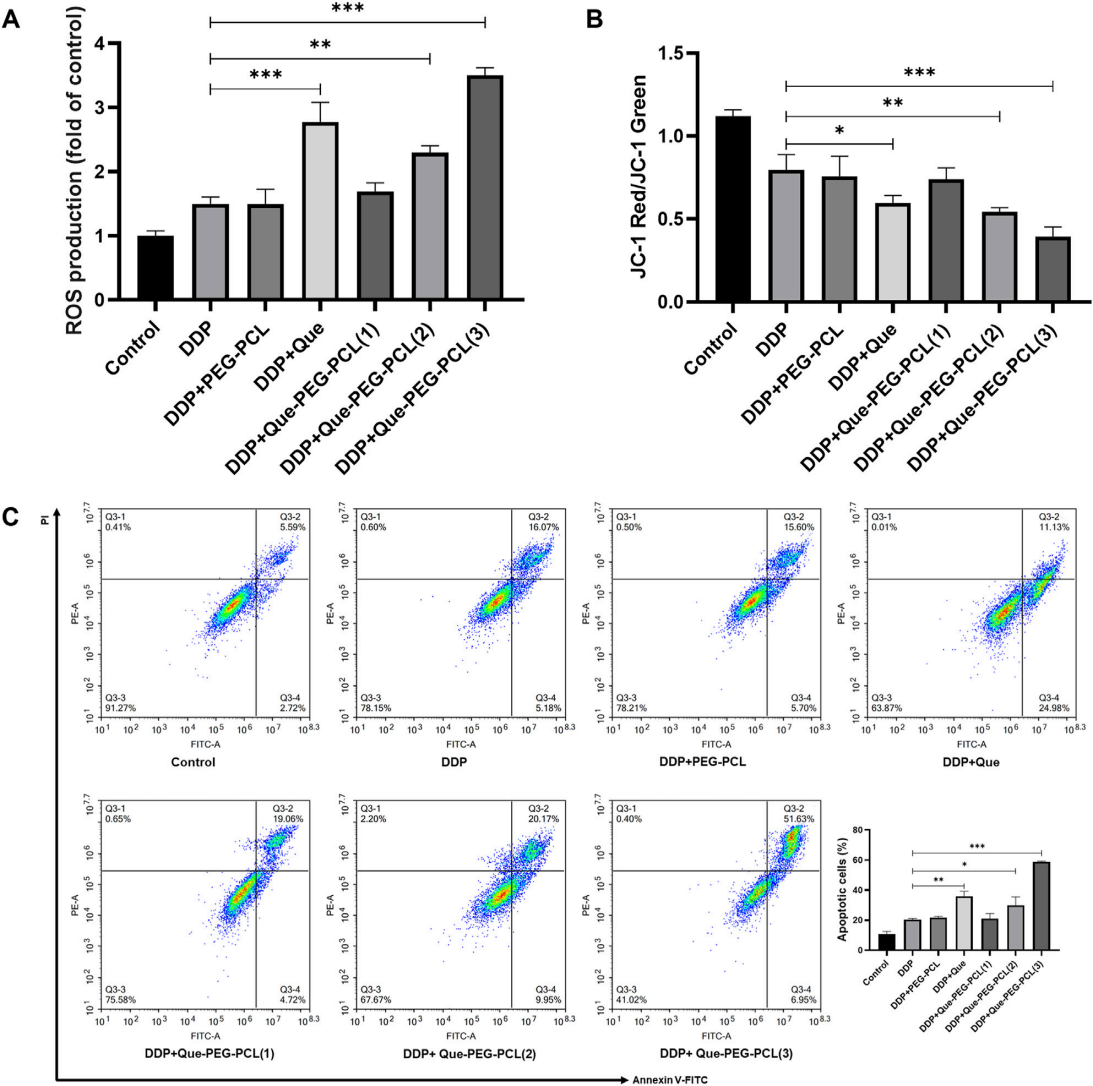
Influence of Que-PEG-PCL on the antitumor activity of DDP in mice with transplanted tumors. **(A)** Reactive oxygen species (ROS) production measured by DCFH-DA fluorescence. **(B)** Mitochondrial membrane potential (ΔΨm) assessed by JC-1 staining (red/green fluorescence ratio). **(C)** Apoptosis rate quantified by flow cytometry (Annexin V/PI staining). Statistical significance is indicated as *p < 0.05, **p < 0.01, ***p < 0.001 compared to the DDP group. Data are expressed as mean ± standard deviation (S.D.), with n = 3.

To quantitatively assess the ROS-generating capacity of various treatment modalities, the fluorescence intensity of dichlorofluorescein (DCF) in cellular samples derived from each treatment cohort were measured. The result revealed that, in comparison to the control group, CT26 cells treated by DDP alone resulted in an increased intensity of green fluorescence, signifying DDP’s -induced intracellular ROS generation. Moreover, the co-application of Que-PEG-PCL with DDP elicited a more pronounced green fluorescence intensity compared to DDP alone, with the peak intensity observed at the maximum concentration of Que-PEG-PCL in combination with DDP. This suggests a synergistic enhancement of ROS production by Que-PEG-PCL (Figure 6A). This finding indicates that Que-PEG-PCL may synergistically enhance the antineoplastic effect of DDP in a dose-dependent manner. Meanwhile, the red/green fluorescence ratio of JC-1 was diminished in the DDP group compared to the control group, signifying DDP-induced mitochondrial membrane potential depolarization. This effect was further attenuated with the cotreatment of DDP and Que-PEG-PCL, manifesting a dose-dependent relationship, with the lowest red/green fluorescence ratio observed in the maximum concentration of Que-PEG-PCL cohort. This suggests that Que-PEG-PCL could potentiate the pro-apoptotic effect of DDP on CT26 cells (Figure 6B). The apoptotic rate within the DDP group was elevated relative to the control group, underscoring the modulatory effect of Que-PEG-PCL on DDP-induced apoptosis. Furthermore, the apoptotic rate in the group cotreated with DDP and Que-PEG-PCL was significantly augmented, with the most pronounced rate observed in the cohort treated with the maximum concentration of Que-PEG-PCL in combination with DDP (Figure 6C). This result underscores the capacity of Que-PEG-PCL to amplify the antineoplastic effect of DDP and to synergistically induce apoptosis in CT26 cells.

### 3.8 Que-PEG-PCL enhances DDP’s antitumor and renoprotective effects in CT26 mice

**FIGURE 7:**
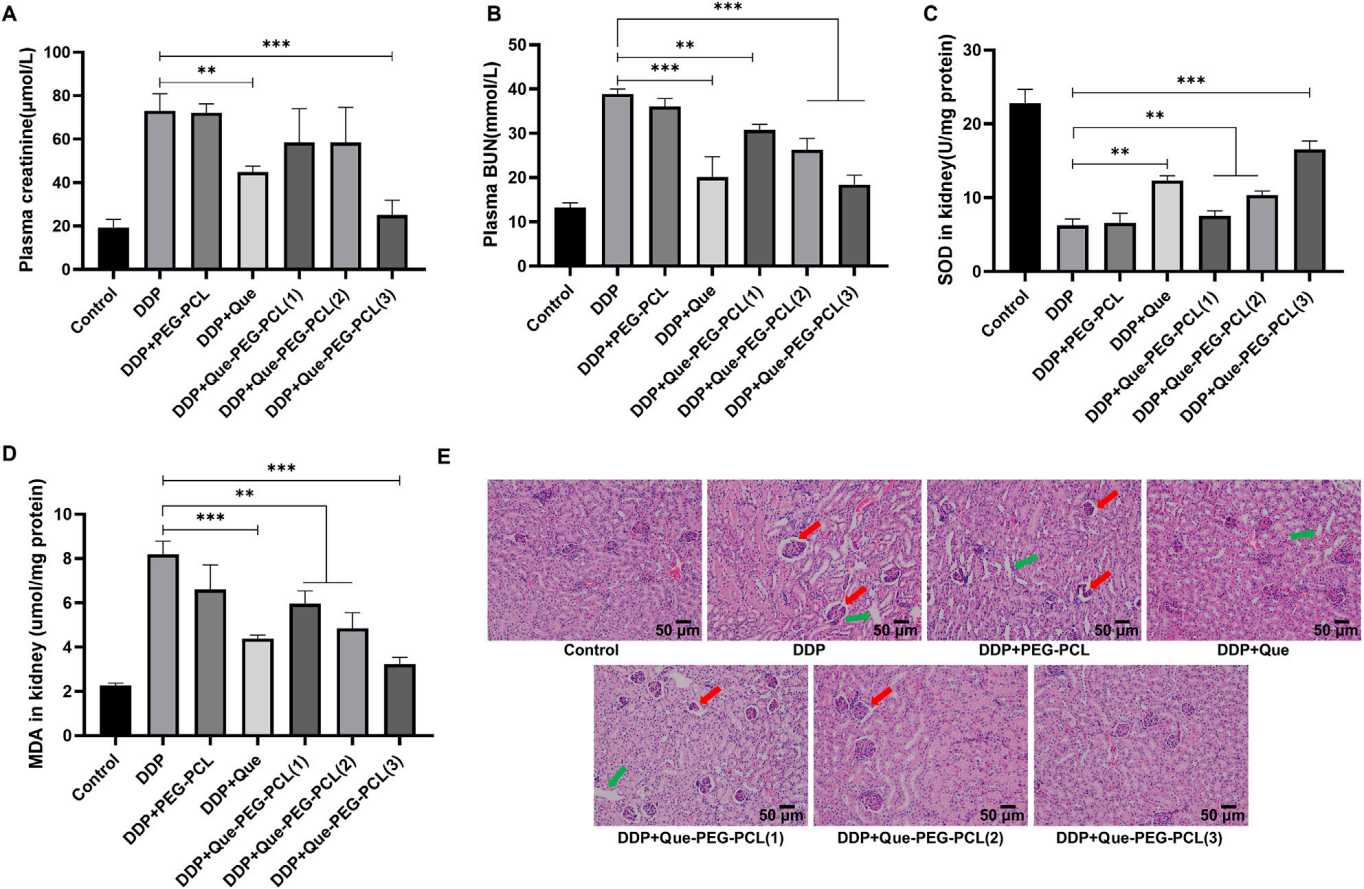
Efficacy of Que-PEG-PCL in conjunction with DDP on renal functionality in tumor-bearing mice. Quantitative analysis of serum Cr **(A)** and BUN **(B)** levels in CT26 tumor-bearing mice subjected to diverse therapeutic interventions. Determination of SOD **(C)** and MDA **(D)** concentrations within renal tissues of CT26 tumor-bearing mice following various treatment regimens. **(E)** Illustrates the histopathological alterations in renal tissues, as assessed by hematoxylin-eosin (HE) staining, in CT26 tumor-bearing mice treated with assorted formulations. Statistical significance is denoted as **p < 0.01 and ***p < 0.001 relative to the DDP group. Data are represented as mean ± standard deviation, n = 3.

To elucidate the dual potential of Que-PEG-PCL micelles in attenuating DDP-induced renal perturbations and augmenting its antineoplastic efficacy, a meticulous evaluation of renal functional parameters was executed in a murine CT26 tumor model. Renal functional impairment was assessed via serum Cr and BUN levels, which are established biochemical indicators of renal function. The cohort administered DDP monotherapy demonstrated a significant elevation in serum Cr and BUN levels relative to the control cohort, signifying pronounced renal toxicity associated with DDP (Figures 7A,B). Conversely, the concurrent administration of DDP and Que-PEG-PCL resulted in a marked reduction of these serum parameters (Figures 7A,B). These findings suggest that Que-PEG-PCL may enhance the antineoplastic activity of DDP while simultaneously mitigates the deleterious effects on renal function induced by DDP therapy. Subsequently, to assess the capacity of Que-PEG-PCL to counteract oxidative stress in the renal tissues of DDP-treated mice bearing CT26 tumors, the levels of SOD and MDA were scrutinized. A diminution in SOD levels and a concomitant escalation in MDA levels were observed in the DDP cohort compared to the control cohort, suggesting heightened lipid peroxidation and oxidative stress. In contrast, the cohort treated with DDP and Que-PEG-PCL exhibited a significant restoration of SOD levels and a reduction in MDA levels, indicating that Que-PEG-PCL effectively attenuates lipid peroxidation and renal injury induced by DDP (Figures 7C,D). Histopathological examination revealed renal tubular distortion, dilation, and vacuolization in the DDP cohort relative to the control cohort. This renal injury was substantially ameliorated by co-treatment with DDP and Que-PEG-PCL (Figure 7E). Hematoxylin and eosin (H&E) staining further corroborated that Que-PEG-PCL not only amplifies the antineoplastic effects of DDP but also markedly ameliorates DDP-induced renal injury, as evidenced by the attenuated severity of histopathological alterations in the treated cohort. Moreover, Histopathological examination of major organs (heart, liver, lung, and spleen) via H&E staining (Supplementary Figure S3) revealed no significant pathological alterations in mice treated with Que-PEG-PCL combined with DDP compared to the control group.

## 4 Discussion

The current study demonstrates that our proposed Que-PEG-PCL effectively mitigate DDP-induced nephrotoxicity while simultaneously enhancing its antitumor efficacy through a dual mechanism involving inhibition of OCT2-mediated renal uptake and potentiation of ROS-dependent apoptosis in tumor cells. These findings address a critical challenge in oncology-the narrow therapeutic window of DDP-by integrating nanomedicine with transporter biology and redox modulation. Our results not only align with prior studies on the nephroprotective role of quercetin and the pharmacokinetic benefits of polymeric micelles but also extend these insights through novel mechanistic and translational discoveries. This study highlights how Que-PEG-PCL combines quercetin’s bioactivity with the delivery benefits of PEG-PCL micelles to achieve synergistic effects. The renal excretion of DDP is characterized by its accumulating in the renal proximal tubules, a key factor underlying DDP-induced nephrotoxicity (Zhang J. et al., 2021). Previous pharmacological investigations have demonstrated that membrane transport proteins mediate the renal excretion of DDP (Nigam, 2015). DDP is known to enter renal tubular cells via OCT2, resulting in renal accumulation and subsequent nephrotoxicity (Freitas-Lima et al., 2020). While free quercetin has been shown to reduce DDP nephrotoxicity through antioxidant and anti-apoptotic pathways (Tang et al., 2023; Oliveira et al., 2024), its clinical utility is limited by poor bioavailability and rapid clearance. By encapsulating quercetin within 30 nm PEG-PCL micelles, we achieved sustained systemic exposure, resolving these pharmacokinetic limitations. The micelles not only preserved renal function by reducing DDP accumulation in proximal tubules (Figures 4A,B) but also enhanced tumor suppression through ROS-mediated apoptosis (Figures 6A–C). This dual functionality-renal protection and tumor sensitization-represents a significant advancement over conventional strategies that often compromise efficacy to mitigate toxicity. Notably, the observed 323% increase in DDP plasma AUC (Table 1) underscores the micelles’ ability to prolong drug circulation, a feature critical for optimizing therapeutic outcomes.

In addition, our findings contrast with prior studies in several key aspects. While earlier work highlighted PEG-PCL’s role in inhibiting the activity of OCT2 (Zhang Y. et al., 2021) or quercetin’s standalone antioxidant (Bi et al., 2022) and antitumor effects (Liu et al., 2021), this study reveals that the micellar carrier itself contributes to OCT2 inhibition (Figures 4E,F). This phenomenon, likely mediated by hydrophobic interactions between the PCL core and transporter domains, amplifies quercetin’s pharmacological activity and introduces a novel mechanism for nanocarrier-mediated transporter modulation. Furthermore, the 84% reduction in tumor weight (Figure 5D) surpasses outcomes reported for free quercetin or PEG-PCL-based chemotherapeutics, underscoring the importance of co-delivering quercetin and DDP to exploit their synergistic pro-oxidant and DNA-damaging effects. These results bridge gaps between nanomedicine and transporter biology, offering a framework for designing multifunctional therapeutics that spatially decouple toxicity and efficacy.

Compared to previous studies, our work provides several unique contributions. For instance, Zhang Y. et al. (2021) only verified that mPEG-PCL could inhibit the activity of OCT2 from the perspective of blank carriers, without investigating the inhibitory effect of drug-loaded preparations on OCT2. Similarly, Bi et al. (2022) only demonstrated the OCT2 inhibitory effect of quercetin monomer *in vitro*, but no reports have shown that quercetin and its drug delivery system can alleviate renal toxicity by inhibiting OCT2 *in vivo*. Our study fills this gap by verifying the regulatory effect of quercetin polymer micelles on the renal excretion process of cisplatin through plasma and urine level changes (Yuan et al., 2023).

In addition, our study provides novel mechanistic insights into the nephroprotective and antitumor effects of Que-PEG-PCL. HEK-293 cells were employed to minimize the influence of physiological condition variations and to better predict effects in humans (Qiu et al., 2018; Bejoy et al., 2022). The significant reduction in renal clearance rate to 0.022 ± 0.003 L/h/kg and the increased survival rate of hOCT2-HEK293 cells after co-administration with Que-PEG-PCL highlight the critical role of OCT2 inhibition in mitigating DDP-induced nephrotoxicity. Oxidative stress and apoptosis are pivotal factors in tumorigenesis and progression (Yu et al., 2022). The loss of mitochondrial membrane potential is a definitive indicator of mitochondrial damage and a hallmark of apoptosis (Zichri et al., 2021). Additionally, the observed increase in ROS levels and mitochondrial membrane potential ratio in CT26 colon cancer cells treated with the combination therapy (Figures 6A–C) further elucidates the pro-apoptotic mechanism underlying the enhanced antitumor efficacy.

The translational potential of our findings is evident from the significant improvements in both renal function and antitumor outcomes in the *in vivo* CT26 colon cancer model. The combination therapy not only reduced tumor weight more effectively than DDP monotherapy but also minimized renal pathological damage, as indicated by lower levels of Cr and BUN in the combination therapy group (Wang et al., 2022). These results suggest that Que-PEG-PCL micelles could serve as a promising platform for clinical applications, offering a synergistic and detoxifying strategy for cisplatin-based chemotherapy.

Future research should focus on addressing the limitations of the current study. Validation in higher-order species or patient-derived xenografts is essential to account for species-specific differences in OCT2 expression and tumor biology. Elucidating the structural basis of PEG-PCL’s interaction with OCT2 using advanced techniques such as cryo-EM or molecular docking could provide valuable insights for designing next-generation nanocarriers. Long-term safety assessments and exploration of combinatorial regimens with immunotherapies are also warranted to further optimize the therapeutic strategy.

## 5 Conclusion

In conclusion, this study redefines DDP therapy by leveraging nanotechnology to concurrently address its nephrotoxicity and enhance its antitumor efficacy. By integrating OCT2 inhibition, ROS potentiation, and tumor targeting into a single platform, we achieve a therapeutic synergy unattainable with conventional approaches. As the first demonstration of dual OCT2/ROS modulation via a single nanocarrier, this research opens new avenues for overcoming the limitations of platinum-based chemotherapy and underscores the transformative potential of flavonoid-based therapeutics in oncology.

## Data Availability

The original contributions presented in the study are included in the article/Supplementary Material, further inquiries can be directed to the corresponding author.
