# Supplementary material for: Molecular pharmacokinetic mechanism of quercetin-encapsulated polymeric micelles in alleviating cisplatin-induced nephrotoxicity and enhancing antineoplastic effects
Source: Front Pharmacol. 2025 Jun 9;16:1590688. doi: 10.3389/fphar.2025.1590688 (PMC12183507; doi:10.3389/fphar.2025.1590688)
Supplement: Supplementary file 1 [file DataSheet1.docx]

Supplementary Material

1. **Supplementary Figures and Tables**
2. **Supplementary Figures**





Fig. S1 Synthesis reaction of PEG-PCL.


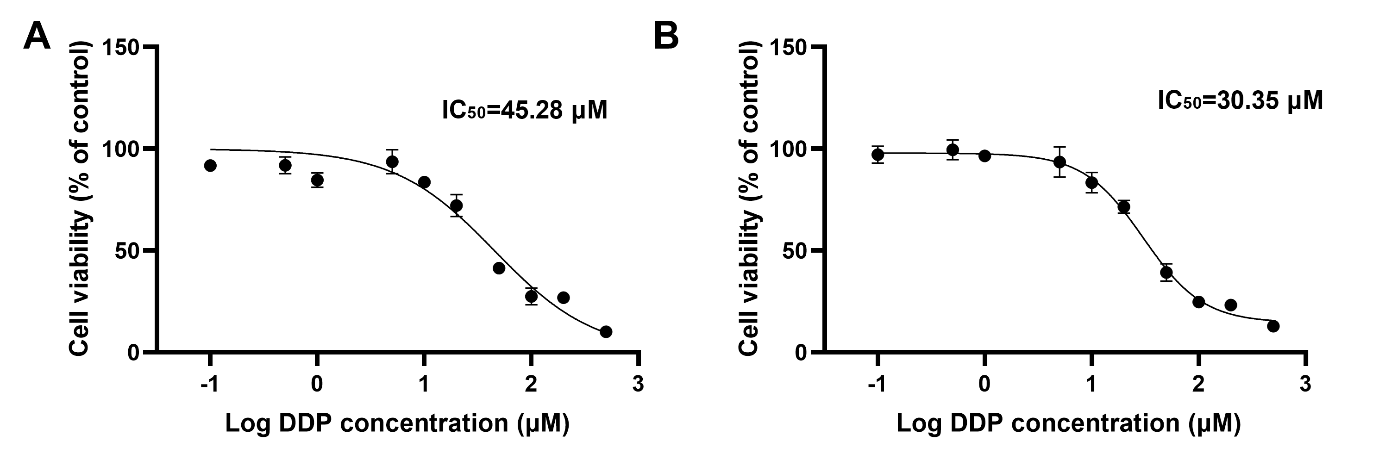


Fig. S2 The cytotoxicities of DDP on Mock-HEK293 **(A)** and hOCT2-HEK293 **(B)** cells.


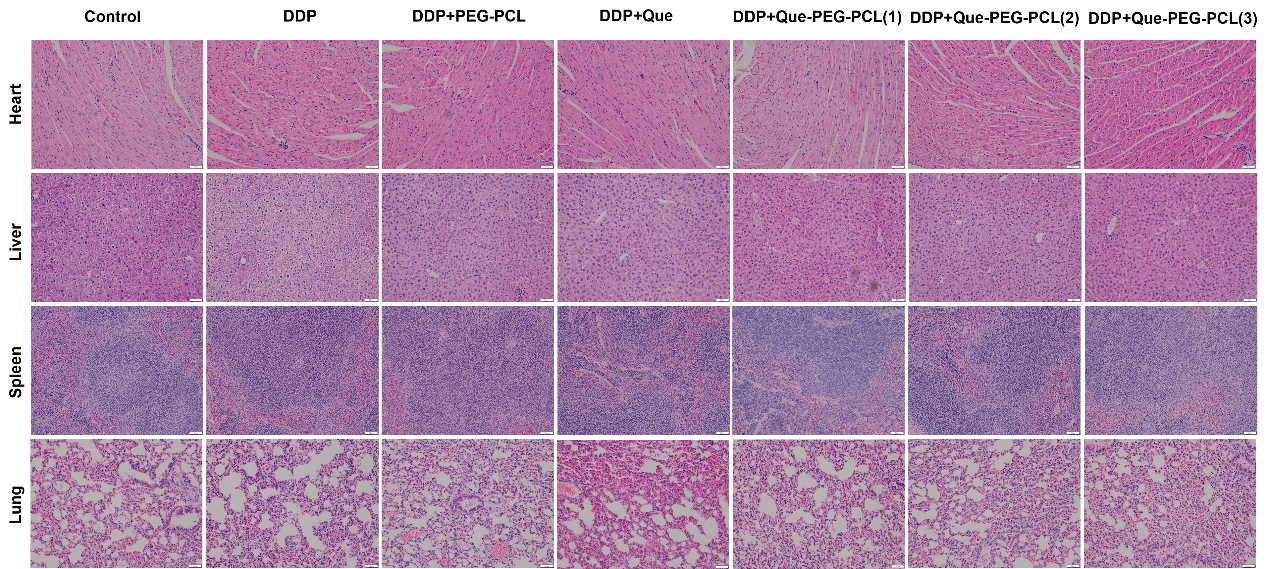


Fig. S3 HE staining of mouse organs in CT26 tumor bearing mice after treatment with diverse preparations.

1. **Supplementary Tables**

**Tab. S1** The molecular weights and polydispersity of PEG-PCL

| Copolymer | Mn | Mw | Mz | Mz/Mw | Mw/Mn |
| --- | --- | --- | --- | --- | --- |
| PEG-PCL | 3947 | 4578 | 5382 | 1.17 | 1.16 |

**Tab. S2** Entrapment efficiency and drug loading of Que-PEG-PCL

| Formulations | EE (%) | DL (%) |
| --- | --- | --- |
| Que-PEG-PCL | 86.77±2.34 | 2.80±0.80 |

**Tab. S3** Particle size, Polydispersity and Zeta potential of PEG-PCL series micelles

| Formulations | Particle size (nm) | Polydispersity | Zetapotential (mv) |
| --- | --- | --- | --- |
| PEG-PCL | 22.53±0.33 | 0.22±0.01 | -1.27±0.85 |
| Que-PEG-PCL | 29.23±3.43 | 0.13±0.01 | -0.08±0.10 |
